# Osteoporosis

**DOI:** 10.1155/2013/952858

**Published:** 2013-10-08

**Authors:** Peng-Fei Shan, Cory J. Xian, Mei Li, Guang-Da Xiang, Ling-Qing Yuan

**Affiliations:** ^1^Department of Endocrinology and Metabolism, Second Affiliated Hospital, Zhejiang University College of Medicine, 88 Jiefang Road, Hangzhou, Zhejiang, China; ^2^Sansom Institute for Health Research and School of Pharmacy and Medical Sciences, University of South Australia, Adelaide, SA 5001, Australia; ^3^Department of Endocrinology, Endocrine Key Laboratory of the Ministry of Health of China, Peking Union Medical College Hospital, Peking Union Medical College, Chinese Academy of Medical Sciences, 1 Shuaifu Road, Beijing, China; ^4^Department of Endocrinology, Wuhan General Hospital of Guangzhou Command, Wuluo Road 627, Wuhan, Hubei, China; ^5^Institute of Metabolism and Endocrinology, the Second Xiang-Ya Hospital, Central South University, 139 Middle Renmin Road, Changsha, Hunan, China

Osteoporosis, a major worldwide public health issue, is characterized by a progressive loss of bone mineral density, disruption of bone microarchitecture, and an increased risk of fractures. Osteoporosis-related fracture causes disability, mortality, and significant financial burden. With the aging of the world population and thus the increasing prevalence, osteoporosis is attracting more and more attention from patients, researchers, clinicians, and government agencies. In recent years, there have been significant advances in our knowledge in bone cell biology, bone metabolism, genetics and pathogenesis of osteoporosis, evaluation of fracture risk of osteoporotic patients, and management of osteoporosis. Our current special issue presents a series of original research and review papers on recent advances in the pathophysiology and clinic aspects of osteoporosis. 

Several studies investigated the relationship between bone turnover biomarkers and bone mineral density (BMD). After examining 2799 individuals aged 20–79 years in Shanghai city of China, W.-W. Hu et al. established normal reference ranges for bone turnover markers (P1NP, OC, and *β*-CTX). They found that these bone turnover markers correlated with BMD. Wu et al. investigated the relationship between the BMD decrease rate and serum levels of OPG, TGF-*β*1, and TGF-*β*2 in 465 healthy Chinese women aged 35–80 years, which showed that TGF-*β*1 is a positive determinant of bone decrease rate. A. Trombetti and colleagues determined the relationship between amenorrhea, IGF-1, and BMD in patients who suffered from anorexia nervosa. Their results suggested that spine BMD is related to hypogonadism, whereas IGF-I predicts proximal femur BMD. Xu et al. found that serum *β*-catenin level is decreased in postmenopausal osteoporotic women compared with postmenopausal non-osteoporotic women. Meanwhile, *β*-catenin is negatively correlated with the expression ratio of RANKL/OPG in the bone.

BMD is the gold standard in osteoporosis diagnosis, and dual X-ray absorptiometry (DXA) is the most widely used tool for BMD measurement. Other methods include quantitative ultrasound, quantitative computed tomography (QCT), peripheral DXA, and other radiographic techniques. N. Li and colleagues compared the osteoporosis detection rates using DXA and QCT. They suggested that QCT may be more sensitive for detecting osteoporosis and may prevent overestimation of BMD by DXA caused by spinal degeneration, aortic calcification, or other sclerotic lesions. 

FRAX is a very popular computer-based algorithm that estimates fracture probability. However, there is no information about the prevalence of fracture risk factors in postmenopausal women who are receiving antiosteoporosis treatment. N. Yurgin et al. demonstrated that multiple risk factors (age >70 years; history of fracture since age 50; minimum reported hip or spine *T*-score ≤−2.5 at diagnosis; body mass index <18.5 kg/m^2^; rheumatoid arthritis; parental history of hip fracture; current cigarette smoking; and oral glucocorticoid use in the 6 months prior to study entry) are associated with a greater incidence of on-study fractures even through the women who have received antiosteoporosis therapies. Their results confirmed that the women with multiple risk factors still remain at an elevated fracture risk even after initiating therapy.

BMD is affected by several factors. In this special issue, some researchers reviewed the relationship between the effect of preterm birth and BMD as well as the impact of age-related changes in trabecular and cortical bone microstructure. To investigate whether foot binding, a historical social custom in China, had effect on BMD, Y. Pan and colleagues demonstrated that foot binding in Southwest China had no influence on lumbar vertebra and femoral neck BMD compared with the unbound control group.

Some drugs used to treat other diseases may have effect on bone metabolism. Y.-J. Wang et al. found that a low dose of testosterone undecanoate (20 mg/d) which is used in elderly male with low serum testosterone could effectively increase lumbar spine and femoral neck BMD. Meanwhile, growth-hormone replacement therapy used in growth hormone deficient adults might have beneficial influence on BMD. H. Wu et al. reviewed the relationship among antipsychotic medication, hyperprolactinaemia, and osteoporosis.

Previously, obesity was thought to have a protective effect on BMD. However, recent studies have suggested an obesity paradox of lower BMD in obese subjects. L. Wang et al. examined the effects of visceral adipose tissue (VAT) and subcutaneous adipose (SAT) tissue on vertebral trabecular bone density using quantitative computed tomography in 320 Chinese women. Their finding suggested that the VAT volume is negatively correlated with BMD in Chinese women aged <55 years old, whilst the SAT volume is not correlated with BMD. Meanwhile, the studies of S.-S. Wu and colleagues demonstrated that omentin, a novel adipokine secreted by visceral adipose tissue, can induce osteoblast proliferation via the PI3K/Akt signaling pathway. Y. Liu et al. reviewed the recent progress in adipokine and bone metabolism. While these studies have provided some clues to explain the relationship between adipose tissue and bone metabolism, further studies are needed to gain a greater appreciation of the mechanisms underlying the effects of obesity on BMD and fracture risk.

Several other basic studies were reported. L. Liu and colleagues reported that puerarin, an isoflavone contained in traditional Chinese herb, could inhibit osteoblast apoptosis through activating estrogen receptor/ERK signal pathway and decreasing Bax/Bcl-2 expression ratio. I. N. Soelaiman et al. revealed that palm tocotrienol, a potent antioxidant, could improve bone histomorphology parameters in overiectomy rats but had no effect on bone biomarkers. Wu et al. successfully established OPG transgenic mouse through microinjection of an OPG vector into fertilized zygotes. They also found that the cancellous and cortical bone volumes and 3D microarchitecture in OPG transgenic mice are significantly improved.

There are still other several interesting articles in this special issue regarding calcium, silico, sex hormone, vitamin D, and bone metabolism as well as bone health. 

The story of osteoporosis is still going on, and our understanding of this disease is advancing. We hope our special issue could bring some latest progress in this filed to the readers. 

